# The Interplay of PPARs with Parasites and Related Intracellular Pathogens

**DOI:** 10.1155/2012/624845

**Published:** 2012-03-01

**Authors:** Marion M. Chan, Dunne Fong

**Affiliations:** ^1^Department of Microbiology and Immunology, School of Medicine, Temple University, Philadelphia, PA 19140, USA; ^2^Department of Cell Biology and Neuroscience, The State University of New Jersey, Piscataway, NJ 08854, USA

The objective of this special issue is to present current findings on how PPARs can tilt the delicate balance between host defense and parasite survival to affect the outcome of parasitic infections. The volume contains five comprehensive reviews and one original research article that describe the mechanisms of action of PPARs on several parasitic diseases which infect differently. The pathogens include the malaria parasite *Plasmodium falciparum* which invades hepatocytes and erythrocytes, the *Leishmania* species which infect macrophages, the intracellular protozoan *Trypanosoma cruzi* which invades all tissues, and the helminth *Schistosoma* species, which live in extracellular environment of specific organs.

Whereas it is well known that many parasitic diseases are exacerbated by the activation of T helper 2, recent research on the anti-inflammatory alternatively activated macrophage (AAM), which is activated by PPAR*γ*, shows that there is another facet to the host immune response [1]. PPAR*γ* primes the differentiation of macrophages towards AAM rather than the proinflammatory classically activated macrophage (CAM) phenotype, by promoting arginase while suppressing inducible nitric oxide synthase expression. This activity favors the survival of pathogens, which may be susceptible to the free radical nitric oxide. On the other hand, PPAR*γ* activation becomes beneficial to the host in malaria. This special issue presents studies that, timely, characterize the interaction between parasites and these macrophages which express the PPAR nuclear factors.

Malaria is the most devastating among diseases of parasitic protozoa. L. Serghides explains how PPAR agonists relieve immunopathology in cerebral malaria. PPAR*γ*-mediated transcription of CD36, a scavenger receptor which enhances phagocytosis and facilitates the removal of parasitized erythrocytes, leads to reduced parasitemia. In addition, the anti-inflammatory action of PPAR*γ* protects the central nervous system from inflammation-mediated destruction. Y. Ren concurs that the strategy to augment the upregulation of CD36 is potentially therapeutic. Using PPAR*γ* agonist rosiglitazone as adjunctive therapy during treatment of cerebral malaria has successfully gone through phase I/IIa trial in Thailand and now awaits a final randomized double-blind placebo-controlled clinical trial.

On the contrary, PPAR activation may benefit the parasite in other infections. For protozoan parasites, trypanosomatids set as examples. M. Chan et al. describe how the activation of PPAR*α* and PPAR*γ* by cutaneous and visceral *Leishmania* species may promote their survival within macrophages. E. Hovsepian et al. also discuss how *Trypanosoma cruzi*-mediated PPAR activation influences intracellular parasite survival. For metazoan parasites, B. Anthony et al. review how the *Schistosoma *species activate PPAR*α*, PPAR*γ*, and AAM to potentiate survival.

The PPAR*γ* agonist rosiglitazone has been shown to possess anti-inflammatory, neuroprotective and neuroregenerative properties. However, although rosiglitazone (drug name Avandia, from GlaxoSmithKline) is still being used to treat type 2 diabetes in the United States, the drug is withdrawn in Europe because of adverse effects, especially to the heart. Pioglitazone may be a better alternative. Extending from rosiglitazone and PPAR*γ*, K. Chen et al. report the protective activity of Wy14643, an agonist of PPAR*α*, the isoform prevalent in the liver, towards hypoxia reoxygenation injury in rodent hepatocytes.

Many parasitic diseases are widespread in developing countries. Historically, they have been regarded as “neglected tropical diseases” [2]. With parasitic infections being increasingly diagnosed in developed countries due to global travel and immigration, their control is being pursued by many investigators [3, 4]. Public and private agencies, such as TDR, Special Programme for Research and Training in Tropical Diseases of World Health Organization, and Bill & Melinda Gates Foundation, using arrangements such as public-private partnerships, are determined to target such diseases of poverty. Scientists from many countries are actively researching various aspects of parasitic diseases. With this background, we sincerely thank the international cast of scientists who have contributed to this special issue on PPARs and parasites. They come from different countries: Argentina, Australia, Canada, China, United Kingdom, and United States, covering the continents Australia, Europe, North, and South America. 

In summary, this special issue provides a glimpse of our contemporary understanding on PPAR involvement in parasitic diseases. Different angles have been explored, for example, while PPAR agonists may decrease immunopathology of cerebral malaria, they may enhance parasite survival in leishmaniasis. We hope the readers will find this special issue of *PPAR Research* informative and will be inspired to make their own contributions to the challenge our world faces from the diverse parasitic infections.
